# Understanding community needs: Comprehensive analysis of the family adoption program conducted in coastal region of South India

**DOI:** 10.1177/22799036261439972

**Published:** 2026-04-22

**Authors:** Ashwini Kumar, Manjula Anil Kunder, Ranjitha S. Shetty, Udyawar Lahari, Bailur Sanjay Kini, Akhila Doddamani, Harshitha Hanglur Narasimha, Kundangar Eshwari, Sneha D. Mallya, Rakshitha R. Shenoy, Afraz Jahan, Divya Arvind Prabhu, Jagnyaseni Maiti

**Affiliations:** 1 Department of Community Medicine, Kasturba Medical College, Manipal Academy of Higher Education, Manipal, India

**Keywords:** Family Adoption Program, medical education, chronic illness, non-communicable diseases, rural healthcare, healthcare utilization, health equity, community-based learning, India

## Abstract

**Background:**

The Family Adoption Program (FAP), launched by the National Medical Commission, is a community-based initiative aimed at enhancing medical student’s understanding of rural health. The program fosters both academic and social impact by aligning with the Sustainable Development Goals. This study aimed to assess the socio-demographic profile, burden of chronic illnesses and healthcare-seeking patterns related to Non-Communicable Diseases (NCD) among families enrolled in FAP in a rural area of South India.

**Design and methods:**

A cross-sectional study was conducted across eight villages in selected taluk of Udupi district, Karnataka chosen by feasibility and convenience sampling. Second-year MBBS students revisited the families they were assigned in their first year. Data were collected using a pre-tested semi-structured questionnaire covering demographics, chronic disease prevalence and healthcare preferences. Analysis was performed using SPSS version16.

**Results:**

Among 696 households surveyed, covering 3364 individuals, 98% response rate, approximately 21.3% reported at least one chronic illness, hypertension (13.9%) and diabetes (10.8%) being the most common. Multivariate analysis showed age more than 45 years (AOR: 13.25, 95% CI: 9.89–17.72), illiterates (AOR: 2.24, 95% CI: 1.48–2.38) as significant independent predictors for the presence of chronic illness. Preference for private healthcare facilities, especially for cancer and cardiovascular diseases, with over 90% of such patients seeking care in the private sector.

**Conclusions:**

FAP not only strengthens student learning through community engagement but also offers critical insights into rural health trends. The study highlights the dual challenge of rising NCD burden and underutilization of public healthcare services in rural regions.

## Introduction

As a significant step toward reshaping medical education, the National Medical Commission (NMC) has introduced key modifications to training, emphasizing community-based learning. One of the most impactful changes is the integration of rural health exposure into the curriculum, requiring medical students to actively engage with villages and address the health concerns of underserved communities.

The Family Adoption Program (FAP) is a community-centered healthcare initiative seamlessly integrated into medical education. It provides medical students with hands-on experience while addressing the healthcare needs of underserved rural populations. Through the program, students are paired with families in rural communities, enabling them to monitor health conditions, deliver preventive care, and build strong communication skills through direct community engagement. By bridging the gap between classroom learning and real-world healthcare delivery, FAP not only enriches medical training but also contributes to better health outcomes in rural areas. It thereby produces socially accountable health care professionals with Quality Education (Sustainable Development Goals (SDG) 4), contributing to health and wellbeing of the rural population (SDG 3).

In 2019, NMC made the FAP a mandatory part of India’s competency-based medical education (CBME) curriculum. The program began implementation with the 2021–2022 academic batch, aligning with national health priorities to increase access to primary care and address healthcare inequities, especially in rural communities.^1,2^

Family adoption program aims to provide an experiential learning opportunity to Indian medical graduates toward community-based health care. It is a program that trains medical students to get a better perspective of the general population residing in a community on various aspects of their health needs and a deeper understanding of the effective and practical approach to address them.

Several studies highlight the benefits of FAP in enhancing student’s clinical abilities and their understanding of the unique challenges of rural health. Shikha et al.^3^ described how FAP could help the students in gaining leadership skills and a deeper understanding of healthcare needs. Similarly, Vanikar and Kumar^4^ emphasize the program’s potential to develop compassionate healthcare providers by fostering long-term relationships between students and the communities they serve. Despite these encouraging findings, limited data exist on the actual health status of the families enrolled under FAP, particularly concerning chronic illnesses and healthcare utilization patterns.

This study was conducted to address this gap by evaluating families adopted by second-year MBBS students. Under the FAP, first-year medical students adopt up to five households in a designated area near their medical college. As part of the program, they continue to follow up with these families during their second and third years of the MBBS course. This study was conducted by MBBS students based on their visits and interactions with the respective families during their second year of medical school. The primary objectives were to assess socio-demographic characteristics, estimate the prevalence of chronic diseases and the pattern of health care utilization for non-communicable diseases. The study also examined the association between chronic illness and key demographic variables such as age and gender. In doing so, it aligns with reduced inequalities (SDG 10), by contributing evidence to support equitable healthcare delivery through medical education initiatives.

## Materials and Methodology

The present cross-sectional study was carried out between February 2023 to December 2023 in Udupi district, Karnataka state. According to National Family Health Survey (NFHS) 5 data, this district in Karnataka is characterized by a high female literacy rate (90.3%), favorable sex ratio of 1148 females/1000 males, which is more than the state average. Despite these advances a major portion of the population resides in rural areas, with a substantial rural-urban divide highlighting the need for equitable health service delivery. The district has a significant coastline along the Arabian Sea, contributing to the local economy through fisheries. It has a total population of 1,177,361 of which 562,799 reside in the selected taluk. The study villages comprised approximately 1600–2000 households. Eight villages from a selected taluk of Udupi district, were selected based on feasibility and convenience.

This community-based survey was conducted in a selected taluk of Udupi district. A house-to-house survey was conducted in the villages such as Katpadi, Kote, Pithrodi, Udyavara, Yenagudde, Vinobha Nagar, Palligudde, Kattegudde, Korangrapadi, Guddeangadi. The survey teams comprised of the faculty members, medical students, medico-social workers and the healthcare workers. Students were organized into groups and taken to the field for data collection according to scheduled timetable. As part of the FAP in accordance with NMC guidelines, each first-year student was assigned a minimum of three households for data collection following a convenience-based approach. All the houses that were adopted in the first Professional year were included in the study. As part of the follow-up visits of FAP, the students visited the same houses in their second year. The designated houses which were locked at the time of the survey were contacted through revisits. The houses that were either untraceable or found door locked at the time of the survey despite repeated revisit attempts were excluded from the study.

**Figure 1.**
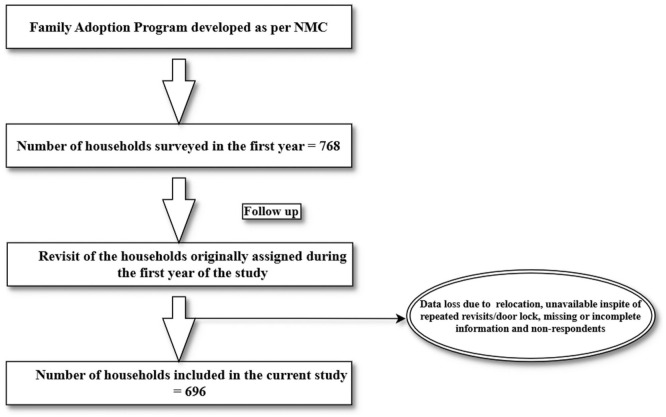
Flow chart illustrating the study process.

Prior to data collection, the students underwent training with standardized instructions on data collection procedures. They were trained to communicate in the local language with the residents and to gather necessary information as per the proforma. Accordingly, 768 households were initially surveyed. During follow up visits, conducted by the students in the second year of their MBBS, the number of eligible households decreased to 710 either due to relocation or door lock. Of the eligible households, 696 consented to participate and provided the required details of the survey yielding a response rate of 98% (Figure 1). The data was subsequently collected using Google forms and the collected data was periodically reviewed by the supervising faculty to ensure completeness and consistency.

Institutional ethical committee [IEC1: 352/2024] clearance was obtained before the start of the study. The permission was obtained from the concerned gram panchayat to visit the localities and adopt the families. Further the ANMs visited the households and obtained verbal consent to collect the data from respected families. Data confidentiality was ensured; no direct identifiers were used. A predesigned, semi-structured validated questionnaire was used which was divided into two sections. Section 1 included household information on socio-economic, demographic, characteristics. Section 2 consists of informant details, anthropometry, blood pressure measurements, presence of chronic illness and source of healthcare availed. The informant from each household, provided comprehensive details of all family members regarding the sociodemographic profile, information on the presence of chronic health conditions and the healthcare sources utilized by affected individuals. The anthropometry measurements were recorded from all the family members who were present at the time of the survey.

The data was checked for accuracy and completeness before carrying out the analysis. The duplicate records were detected using unique household identifiers and were removed after confirmation. Missing data was assessed on the basis of variables and the records with incomplete outcome variables were removed from the relevant analysis. Range checks were performed for numerical values on age, anthropometric indices and blood pressure recordings. The analysis comprised data on 3364 individuals approached from 696 households, whose information was obtained from an adult informant in each house. To obtain additional information related to chronic illness, only family members aged 18 years or older were considered eligible and this data was recorded only among those possessing any supporting medical document.

Height was measured using a flexible narrow non stretch tape to the nearest 0.1 cm by making the subject stand against a wall, faced forward, both feet together, with occiput, scapulae, buttocks and both heels touching the wall and line of vision parallel to the ground. Weight was measured by making the patient stand on a calibrated portable weighing scale. The weight and height was used to calculate Body mass Index (BMI) of the subject. Multimorbidity refers to having two or more long term health conditions at the same time, such as diabetes, heart disease, psychiatric disorder, chronic respiratory illness, thyroid disease, cancer. The information on diagnosed morbidities was based on self-report from the adult participants.

The participants were asked whether the diagnosis has been made by qualified medical practitioner and whether they possessed any supporting medical records such as investigation reports, prescriptions. Blood pressure was measured by the students in the presence of supervising faculty and classified as per JNC 7 classification. Blood pressure was measured using a digital sphygmomanometer with the subject sitting. Three recordings were measured, and the average was used to represent the individuals blood pressure.^2^ The blood pressure was classified as per seventh Joint National Committee (JNC 7) classification. The participants identified as pre-hypertensive were counselled on lifestyle changes and advised to monitor their blood pressure regularly at the nearest healthcare facility. Those with high blood pressure were referred to the nearest government health care facility for further evaluation and management. The individuals with high blood pressure were referred to the nearest government health care facility for management. Similarly, individuals who were overweight or obese were advised to practise lifestyle modifications focussing on diet and physical activity. Waist circumference was measured at the narrowest section of torso, midpoint between the top of the iliac crest and the lower margin of the last palpable rib in the mid axillary line. Hip circumference was measured at a level parallel to the floor, at the largest circumference of the buttocks.

### Statistical analysis

The predictor variables for the study were age (<10 years, between 10 and 89 years was classified with a width of 10, ≥10 years), gender (males, females), marital status (married, unmarried, widow/widower, divorced/ separated, occupation (professional, skilled, semi-skilled, unskilled, homemaker, student, retired and unemployed). The outcome variable considered for the study was the presence of chronic illness (Yes/ No). For further analysis age was re-categorized as ≤45 and >45 years, marital status (married, not living with a partner, education (illiterates, up to SSLC, PUC and above), Occupation (professional, others). The data was entered into Microsoft excel and was analyzed using SPSS version 16.0. Categorical variables were expressed as frequencies and percentages. Quantitative variables were expressed as mean and standard deviation or median and interquartile range depending on the skewness of data. Chi square test was used to test the association of the presence of chronic illness based on key demographic variables. Karl Pearson’s correlation coefficient was used to determine the strength of the relationship between blood pressure recordings and anthropometric indices. Univariate logistic regression was performed to identify factors associated with the presence of chronic illness. Variables with the *p*-value <0.2 in the univariate analysis were included in a multivariable logistic regression model to determine independent predictors using adjusted odds ratio (AOR) and 95% confidence interval. Checks for multicollinearity (VIF < 5) were performed to ensure the robustness of the model. A *p*-value less than 0.05 was considered as statistically significant. The reporting of the study conforms to the STROBE statement (Supplemental File S2).

## Results

In the present study, the team conducted a comprehensive survey encompassing 696 households with a total population of 3364 individuals. Age distribution shows participants across various age groups with the largest proportion between 20 and 49 years (45.3%). Children under 10 years comprised (8.6%) of participants, while 2.3% were aged more than 80 years. The median age among the surveyed individuals was 39 years (IQR: 22–55). Gender distribution was relatively balanced with a slight female (52.1%) predominance over males (47.9%). Educational status varied widely, with (24.4%) having completed secondary education, followed by higher primary (18.1%), PUC/Diploma (17.8%) and graduate/professional degree (17.4%), while (9.8%) were illiterates. Among the 2704 respondents, most were married (66.5%) and a small percentage divorced or separated (0.6%). In terms of occupation, homemakers represented (25.8%), followed by students (21.5%) and professionals (13.8%). Smaller proportions were in other categories including skilled workers (12.5%), unskilled workers (8.9%), semi-skilled workers (8.1%), unemployed individuals (6.6%) and retirees (2.8%). Total of 2717 individuals of age more than 18 years were considered for the estimation of the presence of chronic illness. Among them the median age was identified as 45 years. Around (21.3%) of the individuals were found to have any one chronic illness (Table 1).

**Table 1.** Socio demographic profile of the surveyed population.

Characteristics		Number (%)
Age in years (*n* = 3345)	<10	286 (8.6)
	10–19	435 (13.0)
	20–29	471 (14.1)
	30–39	512 (15.3)
	40–49	533 (15.9)
	50–59	446 (13.3)
	60–69	376 (11.2)
	70–79	211 (6.3)
	80–89	69 (2.1)
	≥90	6 (0.2)
Gender (*n* = 3364)	Females	1754 (52.1)
	Males	1610 (47.9)
Educational qualification (*n* = 3281)	Illiterates	321 (9.8)
	Primary (1–4th Std)	284 (8.7)
	Higher Primary (5–7th Std)	595 (18.1)
	Secondary (8–10th Std)	801 (24.4)
	PUC/Diploma	584 (17.8)
	Graduate/Professional	570 (17.4)
	Postgraduate	126 (3.8)
Marital status (*n* = 2704)	Married	1800 (66.5)
	Unmarried	591 (21.9)
	Widow/Widower	298 (11.0)
	Divorced/Separated	15 (0.6)
Occupation (*n* = 3131)	Professional	432 (13.8)
	Skilled	390 (12.5)
	Semi-skilled	254 (8.1)
	Unskilled	278 (8.9)
	Home maker	807 (25.8)
	Student	674 (21.5)
	Retired	88 (2.8)
	Unemployed	208 (6.6)
Chronic illness^a^ (*n* = 2717)	Yes	579 (21.3)
	No	2138 (78.6)

a Presence of any one chronic illness.

Table 2 presents a comparative analysis of chronic health conditions based on age as well as gender. For 2717 individuals the findings reveal striking disparities in chronic illness prevalence between the age groups. Overall, chronic illness was significantly more common in the older than 45 years group comprising of (41.5%) compared to the less than 45 years (2.6%), p value <0.001. Specific conditions showed similar patterns among individuals of more than 45 years - diabetes affected (21.6%), hypertension (27.9%), cardiovascular disease (3.3%). Chronic respiratory illness and thyroid disorders also showed a similar difference across the age groups. However, psychiatric disorders did not show a significant difference between the age group. All other chronic conditions demonstrated statistically difference between age groups (*p* < 0.05).

**Table 2.** Age and gender wise distribution of chronic illness (*n* = 2717).

		Age (in years)		*p*-Value	Gender		*p*-Value
		≤45 years	>45 years		Males number (%)	Females number (%)	
Illness		Number (%)	Number (%)				
Chronic illness*	Yes	36 (2.5)	543 (41.5)	<0.001	251 (19.6)	328 (22.9)	0.043
	No	1373 (97.4)	765 (58.5)		1030 (80.4)	1108 (77.2)	
Diabetes^b^	Yes	12 (0.9)	282 (21.6)	<0.001	141 (11.1)	153 (10.7)	0.815
	No	1397 (99.6)	1026 (78.4)		1140 (89.0)	1283 (89.4)	
Hypertension^b^	Yes	17 (1.2)	363 (27.9)	<0.001	160 (12.5)	220 (15.3)	<0.001
	No	1392 (98.8)	945 (72.2)		1121 (87.5)	1216 (84.7)	
Cardiovascular disease^b^	Yes	1 (0.1)	43 (3.3)	<0.001	24 (1.9)	20 (1.4)	0.401
	No	1408 (99.9)	1265 (96.7)		1257 (98.1)	1416 (98.6)	
Chronic respiratory illness^b^	Yes	3 (0.2)	21 (1.6)	0.026	10 (0.7)	14 (1.0)	0.737
	No	1406 (99.8)	1287 (98.4)		1271 (99.2)	1422 (99.0)	
Thyroid disorder^b^	Yes	5 (0.4)	22 (1.7)	<0.001	2 (0.2)	25 (1.7)	<0.001
	No	1404 (99.7)	1286 (98.3)		1279 (99.8)	1411 (98.3)	
Cancer^b^	Yes	0 (0.00)	4 (0.3)	-	2 (0.1)	2 (0.1)	0.999^a^
	No	1409 (100.0)	1304 (99.7)		1277 (99.7)	1434 (99.9)	
Psychiatric disorders^b^	Yes	1 (0.1)	3 (0.2)	0.565	1 (0.1)	3 (0.2)	0.698^a^
	No	1408 (99.9)	1305 (99.8)		1280 (99.9)	1433 (99.8)	

a Fishers exact test.

b Includes one or more chronic illness.

* any one chronic illness.

On gender-wise comparison of the prevalence of chronic health conditions. The overall chronic illness shows a slightly higher prevalence among females (22.8%) compared to males (19.6%) and the difference was found to be statistically significant (*p* = 0.043). When examining specific conditions- hypertension and thyroid disorders, showed a significant difference across the genders (*p* < 0.001).

The association between selected socio demographic variables and presence of chronic illness is expressed as unadjusted odds ratio (UOR) and adjusted odds ratio (AOR) with 95% confidence interval (CI). In univariable analysis – age (in years), literacy status, occupation, marital status showed a significant association with the presence of chronic illness. After adjusting for covariates in the multivariable model, age more than 45 years (AOR: 13.25, 95% CI: 9.89–17.72, *p* < 0.01) and illiterates (AOR: 2.24, 95% CI: 1.48–2.38, *p* < 0.01) retained their significance, thereby continuing to predict the presence of chronic illness. No significant association were observed between gender, occupation and marital status after adjusting for confounders as depicted in (Table 3).

**Table 3.** Multivariable analysis to determine the factors associated with presence of chronic illness.

		Total	Presence of chronic illness	UOR (95% CI)	*p*-Value	AOR (95% CI)	*p*-Value
Determinants			Number (%)				
Age (in years)	≤45	1409	60 (4.3)	1	-	1	-
	>45	1308	564 (43.1)	17.04 (12.87-22.57)	<0.001	13.24 (9.89-17.72)	<0.001
Gender	Male	1281	305 (23.8)	1.09 (0.92-1.31)	0.324	-	-
	Female	1436	319 (22.2)	1	-	-	-
Literacy	Illiterate	168	70 (41.7)	5.73 (4.02-8.18)	<0.001	2.24 (1.48-3.38)	<0.001
	Until SSLC	1326	417 (31.4)	3.68 (2.30-4.56)	<0.001	1.85 (1.44-2.39)	<0.001
	PUC and above	1209	134 (11.1)	1	-	1	-
Occupation	Professional	261	32 (12.3)	1	-	1	-
	Others	2368	577 (24.4)	2.31 (1.57-3.38)	<0.001	1.11 (0.71-1.73)	0.650
Marital status	Married	1791	457 (25.5)	1.56 (1.28-1.89)	<0.001	1.13 (0.89-1.44)	0.312
	Not living with a partner	926	167 (18.0)	1	-	1	-

AOR: adjusted odds ratio; UOR: unadjusted odds ratio; 95% CI: 95% confidence interval.

The Table 4 presents the source of healthcare availed by 777 participants with chronic illness. Across all conditions, there is a high preference for private healthcare services over government facilities. This trend is most pronounced for cancer patients, with (100.0%) seeking care from private sources, followed by patients with cardiovascular disease (90.9%). Similarly high proportions of patients with diabetes (86.7%), hypertension (85.5%), and thyroid disorders (85.2%) also opted for private healthcare facilities.

**Table 4.** Source of health care availed for various chronic illness (*n* = 777).^a^

Chronic illness^a^	Illness present	Source of healthcare services	
	Number (%)	Government	Private
		Number (%)	Number (%)
Diabetes	294 (10.8)	39 (13.3)	255 (86.7)
Hypertension	380 (13.9)	55 (14.5)	325 (85.5)
Cardiovascular disease	44 (1.6)	4 (9.1)	40 (90.9)
Psychiatric disorders	4 (0.1)	1 (25.0)	3 (75.0)
Chronic respiratory illness (asthma/COPD)	24 (0.9)	5 (20.8)	19 (79.2)
Thyroid disorders	27 (1.0)	4 (14.8)	23 (85.2)
Cancer	4 (0.1)	-	4 (100.0)

a Includes one or more chronic illness.

The Karl Pearson correlation coefficients (r values) between various anthropometric measures and blood pressure readings, distributed by gender. Among 324 males, age shows a significant positive correlation with systolic blood pressure (SBP; *r* = 0.19, *p* = 0.001). Waist circumference in males demonstrates a significant correlation with SBP (*r* = 0.12, *p* = 0.034). Among 765 females, all anthropometric measures show strong and statistically significant correlations with both systolic blood pressure and diastolic blood pressure (SBP and DBP). Overall, the blood pressure relationship with anthropometric measures appears stronger in females compared to males (Table 5). Sensitivity analysis was not conducted as there were no alternative analytical assumptions or model specifications relevant to the study.

**Table 5.** Correlation between blood pressure recordings and anthropometric indices among the study population.

Variables	Males (*n* = 324)		Females(n = 765)	
	Systolic blood pressure	Diastolic blood pressure	Systolic blood pressure	Diastolic blood pressure
	*r* value (*p*-value)	*r* value (*p*-value)	*r* value (*p*-value)	*r* value (*p*-value)
Age (in years)	0.19 (*p* = 0.001)	0.07 (*p* = 0.207)	0.45 (*p* < 0.001)	0.20 (*p* < 0.001)
Body mass index	0.09 (*p* = 0.104)	0.10 (*p* = 0.069)	0.12 (*p* = 0.001)	0.16 (*p* < 0.001)
Waist circumference	0.12 (*p* = 0.034)	0.05 (*p* = 0.322)	0.13 (*p* < 0.001)	0.11 (*p* = 0.003)
Hip circumference	0.04 (*p* = 0.486)	0.05 (*p* = 0.332)	0.08 (*p* = 0.035)	0.08 (*p* = 0.024)

## Discussion

The data for this study were collected through the Family Adoption Program undertaken by medical students. This study highlights the burden of NCDs in the study population, their distribution across socio-demographic groups as well as the healthcare service utilization across the population. The findings demonstrate the growing prevalence of NCDs, especially amongst the older adult, and dependence on private healthcare facilities. The study also underscores the underutilization of government health services. These patterns reflect characteristics seen across India and within similar rural contexts, highlighting systemic gaps in public health care access.

Our findings indicate that chronic illnesses are significantly more prevalent among individuals above 45 years of age, with hypertension and diabetes being the most common conditions. This pattern aligns with national trends reported in the ICMR-INDIAB study and NFHS-5 data, both of which emphasize the rising burden of NCDs with advancing age in India.^5,6^

The predominance of hypertension among older adults is also consistent with findings from a study conducted by earlier researchers who reported similar age-related increases in cardiovascular risk factors.^7–9^

The current study revealed significant age difference in the prevalence of hypertension. It was found to be significantly higher among older adults (27.9%) than younger adults (1.9%). Gender based difference was seen in the prevalence of hypertension and thyroid. Interestingly we observed the prevalence of hypertension to be higher among females than males. This is in contrast to the earlier studies according to which female older adults had a higher prevalence of hypertension compared to male older adults.^10,11^ The predominance of hypertension among older adults is also consistent with findings from previous studies who reported similar age-related increases in cardiovascular risk factors.^7–9^ Also Prior studies demonstrated that females experience a more rapid progression in blood pressure measurements over their lifetime, suggesting a gender-specific genetic predisposition to hypertension.^12,13^

The prevalence of diabetes mellitus was found to be higher among older adults (21.6%) and was found to be slightly higher among males (11.1%) as compared to females (10.7%). A previous study also observed that diabetes increased with age and is higher among males and more prevalent among older adults.^14,15^

Our results further showed that cardiovascular diseases (3.3%) and thyroid (1.7%) were significantly more prevalent among elderly adults. Beyond age, proportion of cardiovascular diseases was seen more among males (1.9%) as compared to the females (1.4%). Findings from recent studies conducted have also observed comparable findings.^10,15–18^

One notable aspect of our study is the trend on health care utilization which showed a overwhelming preference for private healthcare services among people with chronic conditions. Over 86.7% of people with diabetes, 85.5% high blood pressure, 90.9% heart disease and 85.2% thyroid issues sought treatment in private facilities, while all cancer patients used private healthcare. This finding highlights deep seated inequities in service use. Concerns over cost and financial hardship are raised by this tendency, especially for groups that are already economically weak. Prior study similarly reports low engagement with government funded health schemes despite their availability.^19^

The preference for private healthcare may be attributed to perceived better quality of services, shorter waiting times and access to specialists, as noted in the Kerala Healthcare Utilization Report.^20^ Recent study demonstrates that although public health institutions offer NCD services, utilization by public is still low because of accessibility barriers and a lack of awareness.^21^

Additionally, the present study found correlation between anthropometric findings and blood pressure. Interestingly this relationship was found to be highly significant among females. As seen in a recent study, our analysis also showed a significant positive correlation between age and SBP in both genders.^22^Among females, BMI, waist circumference and hip circumference were significantly associated with both SBP and DBP, which supports previous findings underscoring central obesity as a critical predictor of hypertension in South Asian women.^23^ A recent study by Shenoy et al. reported that lower educational attainment emerged as a significant determinant for chronic illness. This aligns closely with the findings of the present study suggesting limited education may influence health outcomes.^24^

The study comprehensively examined both socio-demographic characteristics and chronic disease patterns, enabling analysis of associations between these variables. Strengthening health awareness around NCD services at public health care institutions and government insurance schemes is crucial. Involving medical students in data collection not only enriched their community health exposure but also ensured wide geographic and population coverage generating valuable surveillance data. Further, the study provides valuable information about demographic and anthropometric risks, targeted interventions for NCD – especially among older adults and women. Emphasizing central obesity control through diet and exercise can reduce hypertension rates. Latest findings suggest that the stronger correlations in females highlight the need for gender-sensitive preventive interventions focusing on weight management and lifestyle modifications.^25,26^

## Strengths of the study

The present study conducted within the NMC mandate guidelines on FAP provides community-based evidence on the burden of chronic disease in the surveyed population. The survey with standardized training of medical students along with faculty supervision and systematic data quality checks strengthens the relevance of the findings. Involving medical students in data collection not only enriched their community health exposure but also ensured wide geographic and population coverage and comprehensively examined both socio-demographic characteristics and chronic disease patterns, enabling analysis of associations between these variables. Further, the study provides valuable information for early identification of at-risk groups, supporting future planning and targeted interventions for NCDs.

## Limitations of the study

The selection of the villages and households were based on feasibility and convenience the findings may be subject to possible selection bias which may limit generalizability beyond similar settings. Sample size was set as per the programmatic mandate governed by NMC requirement on allocation of household to the students. Hence, it may affect the generalizability of the result despite the high response rate. Chronic disease status was primarily based on self-reports, which may have led to under or over reporting of conditions although efforts were made to verify the diagnosis using available medical records. Health care preference, perceived quality was not directly assessed and interpretations regarding public-private care utilization are based on patterns observed and supporting literature.

## Conclusion and recommendations

The preference for private health care highlights gaps in public service utilization. Gender specific association between anthropometry and blood pressure highlights the need for targeted, preventive strategies and reinforce the need for integrating community engagement into medical education. This would not only improve student learning but also help to generate real world data that may guide targeted public interventions.

## Supplemental Material

sj-docx-1-phj-10.1177_22799036261439972 – Supplemental material for Understanding community needs: Comprehensive analysis of the family adoption program conducted in coastal region of South India Supplemental material, sj-docx-1-phj-10.1177_22799036261439972 for Understanding community needs: Comprehensive analysis of the family adoption program conducted in coastal region of South India by Ashwini Kumar, Manjula Anil Kunder, Ranjitha S. Shetty, Udyawar Lahari, Bailur Sanjay Kini, Akhila Doddamani, Harshitha Hanglur Narasimha, Kundangar Eshwari, Sneha D. Mallya, Rakshitha R. Shenoy, Afraz Jahan, Divya Arvind Prabhu and Jagnyaseni Maiti in Journal of Public Health Research

sj-docx-2-phj-10.1177_22799036261439972 – Supplemental material for Understanding community needs: Comprehensive analysis of the family adoption program conducted in coastal region of South India Supplemental material, sj-docx-2-phj-10.1177_22799036261439972 for Understanding community needs: Comprehensive analysis of the family adoption program conducted in coastal region of South India by Ashwini Kumar, Manjula Anil Kunder, Ranjitha S. Shetty, Udyawar Lahari, Bailur Sanjay Kini, Akhila Doddamani, Harshitha Hanglur Narasimha, Kundangar Eshwari, Sneha D. Mallya, Rakshitha R. Shenoy, Afraz Jahan, Divya Arvind Prabhu and Jagnyaseni Maiti in Journal of Public Health Research
