# Supplementary material for: Understanding community needs: Comprehensive analysis of the family adoption program conducted in coastal region of South India
Source: J Public Health Res. 2026 Apr 22;15(2):22799036261439972. doi: 10.1177/22799036261439972 (PMC13111829; doi:10.1177/22799036261439972)
Supplement: sj-docx-1-phj-10.1177_22799036261439972 – Supplemental material for Understanding community needs: Comprehensive analysis of the family adoption program conducted in coastal region of South India [file sj-docx-1-phj-10.1177_22799036261439972.docx]

Family Adoption Program

Name of student:

Registration number of student:

Mobile number of student:

Name of mentor:

Signature of mentor:

Signature and name of the staff who accompanied for family visit:

Village: Locality: Head of the family: Full postal address (including house number):

Respondent’s Mobile number: Panchayat:

Date of visit :

**Section -1**

**Informant details:**

**Name :**

**Age : ______ Years**

**Gender: Male/Female/Others**

**Details of family members:**

| **Sl. No.** | **Name** | **Relation to the head of the family** | **Age** | **Gender** | **Marital Status** | **Educational Status** | **Occupation** |
| --- | --- | --- | --- | --- | --- | --- | --- |
|  |  |  |  |  |  |  |  |
|  |  |  |  |  |  |  |  |
|  |  |  |  |  |  |  |  |
|  |  |  |  |  |  |  |  |
|  |  |  |  |  |  |  |  |
|  |  |  |  |  |  |  |  |
|  |  |  |  |  |  |  |  |
|  |  |  |  |  |  |  |  |

**Total family income:**

**Section-2**

**CHRONIC ILLNESS**

Does any family member currently suffer from any of the illnesses? (Details to be collected for all members even if they are away for work or any other reason)

***Eg: Diabetes mellitus, Hypertension, Cardiovascular disease, Asthma,*** Any other, specify.

If a person has more than one illness, enter each illness one below other sequentially in one row only.

| Sl. No | Name | Age | Sex | Illness | Duration of illness | On regular treatment  Yes/no/not aware | Source of health care * |
| --- | --- | --- | --- | --- | --- | --- | --- |
| 1. |  |  |  |  |  |  |  |
| 2. |  |  |  |  |  |  |  |
| 3. |  |  |  |  |  |  |  |
| 4. |  |  |  |  |  |  |  |

*Private practitioner clinic (1), Govt district hospital (2), KMC hospital (3), Pvt. Hospital (4), Primary/Community health center(5), Sub centre(6), AYUSH(7), Others(Specify)(8)

# DETAILS OF HOSPITALIZATION

**Give details of any hospitalization in the family in last one year including delivery. (document latest TWO if many)**

**# Place of admission**: Government/Private

*Type of treatment: 1. Medical 2. Surgical 3. Other (specify)

****Outcome**: 1.Discharged/ 2.Referred to higher centre/3.Death/4.Disability/ 5.Other (Specify)

*****Source of funding**: 1.Salary/ 2.Savings/3.Borrowing/ 4.Sale of assets/5.Employer insurance/6.Personal insurance/ 7.Manipal AarogyaSuraksha card)/8.Pension/9.Free of cost/10.Self-employed business/11. ESI/ 12. Aayushman Bharat/13. Any other (specify)

**Hospitalization 1**

**Name Age/gender**

# Condition for which admission was done

# Place of admission^#^

# Duration of hospitalization (in days) Type of treatment*

**Outcome****

# Total expenditure (Rs.) Source of funding*** Hospitalization 2

**Name Age/gender**

# Condition for which admission was done:

**Place of admission^#^**

# Duration of hospitalization (in days) Type of treatment*

**Outcome****

# Total expenditure (Rs.) Source of funding***

**ANTHROPOMETRY AND BLOOD PRESSURE MEASUREMENT (for adults)**

| **Sl. No** | **Name** | **Age** | **Sex** | **Height (cm)** | **Weight** | **BMI**  **(kg/m^2^)** | **Waist circumference (cm)** | **Hip circumference (cm)** | **Waist Hip Ratio** | **BP** |
| --- | --- | --- | --- | --- | --- | --- | --- | --- | --- | --- |
| **1** |  |  |  |  |  |  |  |  |  |  |
| **2** |  |  |  |  |  |  |  |  |  |  |
| **3** |  |  |  |  |  |  |  |  |  |  |
| **4** |  |  |  |  |  |  |  |  |  |  |
| **5** |  |  |  |  |  |  |  |  |  |  |
| **6** |  |  |  |  |  |  |  |  |  |  |
